# Insights into Antifungal Mechanisms of *Bacillus velezensis* S141 against *Cercospora* Leaf Spot in Mungbean (*V. radiata*)

**DOI:** 10.1264/jsme2.ME22079

**Published:** 2023-03-18

**Authors:** Pongpan Songwattana, Pakpoom Boonchuen, Pongdet Piromyou, Jenjira Wongdee, Teerana Greetatorn, Sukanya Inthaisong, Piyada Alisha Tantasawat, Kamonluck Teamtisong, Panlada Tittabutr, Nantakorn Boonkerd, Neung Teaumroong

**Affiliations:** 1 School of Biotechnology, Institute of Agricultural Technology, Suranaree University of Technology, 30000, Thailand; 2 School of Crop Production Technology, Institute of Agricultural Technology, Suranaree University of Technology, Nakhon Ratchasima, 30000, Thailand; 3 The Center for Scientific and Technological Equipment, Suranaree University of Technology, Nakhon Ratchasima 30000, Thailand

**Keywords:** *Bacillus velezensis*, *Cercospora* leaf spot, biocontrol agent, antifungal activity, transcriptome ana­lysis

## Abstract

*Cercospora* leaf spot (CLS) is caused by *Cercospora canescens* and is one of the most important diseases of mungbean (*Vigna radiata*). *Cercospora* leaf spot may result in economic loss in production areas. The present study investigated the potential of *Bacillus velezensis* S141 as a biocontrol agent for *C. canescens* PAK1 growth on culture plates. Cell-free secretions from a dual culture of S141+PAK1 inhibited fungal growth more than those from a single culture of S141. The biocontrol efficiency of S141 against *Cercospora* leaf spot on mungbean was then evaluated by spraying. The disease severity of *Cercospora* leaf spot was significantly reduced in plants treated with S141, with a control efficiency of 83% after 2 days of infection. Comparative transcriptomics and qRT-PCR ana­lyses of S141 during *C. canescens* inhibition were performed to elucidate the antifungal mechanisms underlying its antifungal activity against *Cercospora* leaf spot. According to the differentially expressed genes, most up-regulated genes involved in the biosynthetic genes encoding enzymatic hydrolases, including protease, β-glucanase, and N-acyl glucosaminase, were detected in strain S141 following its interaction. Moreover, genes related to secondary metabolites (surfactin, bacilysin, and bacillomycin D) were up-regulated. Collectively, these results suggest that S141 exhibited strong antifungal activity against *C. canescens* due to multiple enzymatic hydrolases and secondary metabolites. Therefore, the present study provides insights into the biological network responsible for the antifungal activity of *B. velezensis* S141 against *C. canescens*.

*Cercospora* leaf spot (CLS) is caused by *Cercospora canescens* and is a serious disease in mungbean (*Vigna radiata*). It may result in significant yield losses in production areas (Iqbal *et al.*, 1995; Chand *et al.*, 2012). *Cercospora* leaf spot often develops under high humidity conditions in irrigated fields at a temperature of approximately 25–35°C in the daytime and higher than 16°C at night (Kumar *et al.*, 2011). Various control strategies are employed to manage *Cercospora* leaf spot, including chemical treatments and resistant mungbean cultivars. However, the use of chemical treatments in the field may have a negative impact, such as environmental pollution and health hazards. Therefore, biological control methods using micro­organisms are a promising alternative.

The genus *Bacillus* sp. is now widely used as a biological control agent to suppress various plant diseases. Some species also have beneficial effects on plant promotion (Borriss, 2011; Chen *et al.*, 2020). For example, *B. amyloliquefaciens* strain GKT04 exhibited antifungal activity against *Fusarium* wilt in banana (Tian *et al.*, 2021). Many strains of *Bacillus* species are efficient biological agents that control the growth of *Fusarium* wilt disease, *e.g.*, *B. velezensis* (Jiang *et al.*, 2019), *B. subtilis* (Chen *et al.*, 2017), and *B. aneurinilyticus* and *B. firmus* (Abed *et al.*, 2016). However, only a few biological methods are currently used to manage *Cercospora* leaf spot in mungbean. The biocontrol activities of *Bacillus* strains are mediated by the compounds secreted as a result of enzymatic hydrolysis and/or secondary metabolites. The efficiency of biological control by different microorganisms is controlled by different mechanisms (Harwood *et al.*, 2018). In addition to hydrolytic enzymes and secondary compounds, other biological aspects, including quorum sensing, biofilm formation, and bacterial motility functions, are regarded as important properties for biological control (Leclère *et al.*, 2006; Khoiri *et al.*, 2017; Chen *et al.*, 2018; Albarracín Orio *et al.*, 2020).

*B. velezensis* S141 is an effective plant growth-promoting rhizobacteria (PGPR) isolated from soybean rhizosphere soil in Thailand (Prakamhang *et al.*, 2015). The whole-genome sequence of S141 has been published (Sibponkrung *et al.*, 2017). A co-inoculation with S141 and soybean-nodulating bradyrhizobia (*B. diazoefficiens* strains USDA110 and THA6) significantly improved soybean growth and yields in field experiments (Sibponkrung *et al.*, 2020). Moreover, S141 inhibited the mycelial growth of *C. canescens*. Its ability to promote plant growth and nodulation efficiency in a *Bradyrhizobium* co-inoculation has already been demonstrated (Prakamhang *et al.*, 2015; Sibponkrung *et al.*, 2020). However, the antifungal potential of S141 remains unclear. To elucidate the mechanism of action of S141 against *C. canescens*, the present study attempted to obtain transcriptomic insights into the antifungal activity of *B. velezensis* S141. The efficacy of biocontrol by S141 to suppress *Cercospora* leaf spot disease in mungbean (*V. radiata* CN72; cell line susceptible to CLS) was also evaluated. The present study describes the biological network responsible for the antifungal activity of *B. velezensis* S141 against *C. canescens*.

## Materials and Methods

### Bacterial strains and growth conditions

*C. canescens* (PAK1) was purchased from Prof. Tantasawat, School of Crop Production Technology, Suranaree University of Technology. It was cultured on potato dextrose agar (PDA) at 30°C to generate mycelia. *B. valezensis* S141 was cultivated in LB (Luria Bertani broth) at 180‍ ‍rpm at 30°C for 24 h.

### *In vitro* antifungal assays

The evaluation of antifungal activity by S141 against *C. canescens* (PAK1) was assessed using dual culture assays. Briefly, a 0.5-cm mycelial plug taken from the edge of actively growing colonies of PAK1 was placed on the center of a PDA plate and incubated at 30°C for 10 days. A S141 colony was then placed approximately 3‍ ‍cm from this colony using an inoculation loop. Plates were incubated at 30°C. A PDA plate culture containing only a PAK1 mycelial colony served as the control. Antagonistic effects were evaluated via the colony morphology of *Cercospora* fungi versus the control plate. We recorded the percentage of mycelial growth inhibition after an incubation at 30°C for 14 days. The diameters (cm) of fungal colonies on plates from control and dual cultivations were calculated using the following formula:

Mycelial growth inhibition (%)=([cd–td]/cd)×100

where cd=diameter of the control plate and td=diameter of the dual culture plate.

A dual culture of S141 and PAK1 mycelia was performed to examine the effects of S141 on *Cercospora* mycelial proliferation. PAK1 mycelia were prepared from a PDB liquid culture on a rotating shaker (50‍ ‍rpm) at 30°C for 7 days. Approximately 100‍ ‍mg of mycelia was transferred into fresh PDB and inoculated with 1% of the S141 culture (10^8^ CFU mL^–1^). A liquid culture of mycelia without S141 was used as the control. The experiment was conducted on a rotating shaker (50‍ ‍rpm) at 30°C. Antifungal activity was assessed by the mycelial dry weight (mg) of *Cercospora* fungi versus the control culture (PAK1 single culture). Mycelia were collected by filtering with miracloth (22–25‍ ‍μm) (MilliporeSigma) and drying at 65°C for 48 h. The percentage of mycelial growth inhibition was assessed 24 and 48‍ ‍h after the S141 inoculation using the following formula:

Mycelial growth inhibition (%)=([cMDW–tMDW]/cMDW)×100

where cMDW=mycelial dry weight of the control culture and tMDW=mycelial dry weight of the dual culture.

To evaluate the antifungal activity of bacterial secretions produced by S141 against *Cercospora* fungi, PAK1 mycelia were cultured in the secretions from a single culture of S141 and dual culture of S141 combined with PAK1 fungi. The secretions of S141 (SC-S141) and the dual culture (SC-S141+PAK1) were collected from a 48-h culture in PDB medium using centrifugation (4,000‍ ‍rpm, 20‍ ‍min) and 0.2-μm filtration. PAK1 mycelia were cultured in secretions on a rotating shaker (50‍ ‍rpm) at 30°C for 24 h. The antifungal activity of secretions was assessed via the percentage of mycelial growth inhibition (as described above). Growth inhibition was compared with that by the dual culture (S141 cells+PAK1). The culture of PAK1 mycelia in PDB was used as the control. All *in vitro* antifungal assays were performed using five replicates.

### Scanning electron microscopy (SEM)

PAK1 mycelia from the single and dual cultures of S141+PAK1 were collected by filtering with miracloth (22–25‍ ‍μm) (MilliporeSigma) after 24 and 48‍ ‍h of cultivation. The mycelia collected were fixed with 2.5% glutaraldehyde (Electron Microscopy Sciences) in 0.1 M phosphate buffer (pH 7.2) at 4°C overnight, washed three times in phosphate buffer (4°C, pH 7.2), fixed in 1% osmium tetroxide (Electron Microscopy Sciences) for 2 h, washed three times in distilled water (room temperature), and then dehydrated in a graded acetone series (20, 40, 60, 80, and 100% three times). Specimens were dried in a critical point drying apparatus using liquid carbon dioxide as the medium. Hydrated specimens were affixed with conductive silver paint and coated with gold palladium (thickness ~15‍ ‍nm). Samples were imaged with a field emission scanning electron microscope (FESEM_ZEISS model-Auriga, Coater _ Leica EM ACE600).

### Evaluation of biocontrol by S141 on mungbean Cercospora leaf spot

Mungbean seeds (*V. radiata* cv. CN72) were sterilized with 95% alcohol for 30‍ ‍s and 3% sodium hypochlorite for 5‍ ‍min and were then rinsed 10 times with sterilized water. Germinated seeds were planted in a Leonard’s jar system filled with sterilized vermiculite (Somasegaran and Hoben, 2012) and maintained with BNM medium containing 5‍ ‍mM NH_4_NO_3_ (Ehrhardt *et al.*, 1992). Plants were grown under controlled environmental conditions of 28±2°C on a 16-h light/8-h dark cycle at a light intensity of 300 μE m^–2^ S^–1^ and 50% humidity. After three weeks, the S141 cell suspension (10^6^ CFU mL^–1^) in BNM medium was sprayed on leaves. Treated (with S141 spraying) and non-treated plants (without S141 spraying) plants were inoculated with the PAK1 mycelial suspension via the dropping method. Briefly, a mycelial suspension was prepared from 5‍ ‍mL of the mycelial suspension containing 80 mycelial plugs (5.5-mm cork bore) taken from the edge of actively growing colonies of PAK1 on PDA. Four drops of the mycelial suspension (5‍ ‍μL drop^–1^) were placed on the bottom of a leaf after plants had been irrigated 1‍ ‍d before the inoculation to maintain high humidity for proper pathogenesis and disease development. *Cercospora* leaf spots on infected leaves was scored on a scale of 1 to 5 for symptom expression (Chankaew *et al.*, 2011), and these scores were used in assessments of the percentage of the disease severity index (%DSI) using the following formula:

Disease severity index (%)=(sum of scores in treatment/[Total number of observations in treatment]×[maximal score value])×100

### Transcriptome ana­lysis of S141 during C. canescens PAK1 inhibition

#### RNA extraction and sequencing

To obtain samples for RNA sequencing, S141 cells from the single culture (S141) and dual culture (S141+PAK1) were collected 24‍ ‍h after cultivation. In the single culture of S141, 1% of the S141 culture (OD_600_=1; 10^8^ CFU mL^–1^) was added to fresh PDB. In the dual culture of S141+PAK1, approximately 100‍ ‍mg of mycelia was transferred into fresh PDB and inoculated with 1% of the S141 culture. Cultures were then incubated on a rotating shaker (50‍ ‍rpm) at 30°C for 24 h. S141 bacterial cells from the single culture were collected by the centrifugation method (4,000‍ ‍rpm, 15‍ ‍min). In the dual culture, PAK1 mycelia were removed by filtering with miracloth (22–25‍ ‍μm) (MilliporeSigma) before the collection of S141 cells as described before. The total RNA of S141 from the single and dual cultures was isolated using the FavorPrep Tissue Total RNA Mini Kit (Favorgen) according to the manufacturer’s instructions. The total RNA of each sample was measured with an Agilent 2100/2200 Bioanalyzer (Agilent Technologies), NanoDrop (Thermo Scientific), and 1% agarose gel; 1‍ ‍μg total RNA was used for library preparation. Next-generation sequencing (NGS) library preparations were constructed from RNA combinations using three replicate RNA samples of each treatment according to the manufacturer’s protocol. Libraries with different indices were then multiplexed and loaded on an Illumina HiSeq instrument according to the manufacturer’s instructions (Illumina). Illumina sequencing was performed by Azenta Life Sciences using the paired-end (PE) 150 configuration.

### Differentially expressed genes (DEGs) and gene ontology (GO) enrichment ana­lyses

A differential expression ana­lysis was performed using the DESeq2 Bioconductor package, which is a model based on a negative binomial distribution. After adjustments via Benjamini and Hochberg’s approach to control the false discovery rate, the significance of DEGs was evaluated under the criteria of *P*<0.05 and log_2_FC >1 or <–1. The GO classification of up-regulated (*P*<0.05, log_2_FC >1) and down-regulated genes (*P*<0.05, log_2_FC <–1) used a comparison with S141 during PAK1 inhibition.

### Differential gene expression ana­lysis of S141 during *Cercospora* inhibition

To examine differential gene expression in S141 during PAK1 inhibition, S141 cells from the single and dual cultivations were harvested at different time intervals from 0 to 48 h. S141 cell suspensions in PDB were adjusted to 0.5 (OD_600_) (10^6^‍ ‍cells‍ ‍mL^–1^) at the beginning of the cultivation. PAK1 mycelia were then added to the S141 culture and incubated on a rotating shaker (50‍ ‍rpm) at 30°C. S141 cells were collected at 0, 12, 24, 36, and 48‍ ‍h by centrifugation. The single cultivation of S141 was the control. Total bacterial RNA was extracted as described above. Contaminated DNA was removed by RNase-free DNase I (Thermo Scientific) according to the manufacturer’s instructions. The synthesis of cDNA was performed using a high-capacity cDNA reverse transcription kit (Bio-Rad according to the manufacturer’s protocol. cDNA was subjected to PCR amplification using gene-specific primers (Table S1) designed from representative up- and down-regulated DEGs. PCR amplification was performed using the Luna^®^ Universal qPCR Master Mix (New England Biolabs) with the following PCR program: an initial denaturation step at 95°C for 3‍ ‍min followed by PCR cycling at 95°C for 30‍ ‍s and 60°C for 30‍ ‍s for 40 cycles. Relative gene expression was calculated using the comparative Ct (2^–ΔΔCT^) method (Livak and Schmittgen, 2001) and normalized to the expression of 16S rRNA (Table S1). Expression data from biological triplicates in two independent experiments were evaluated.

## Results

### Potential antifungal activity of B. velezensis S141 against *Cercospora* leaf spot *disease*

**Fig. 1.**
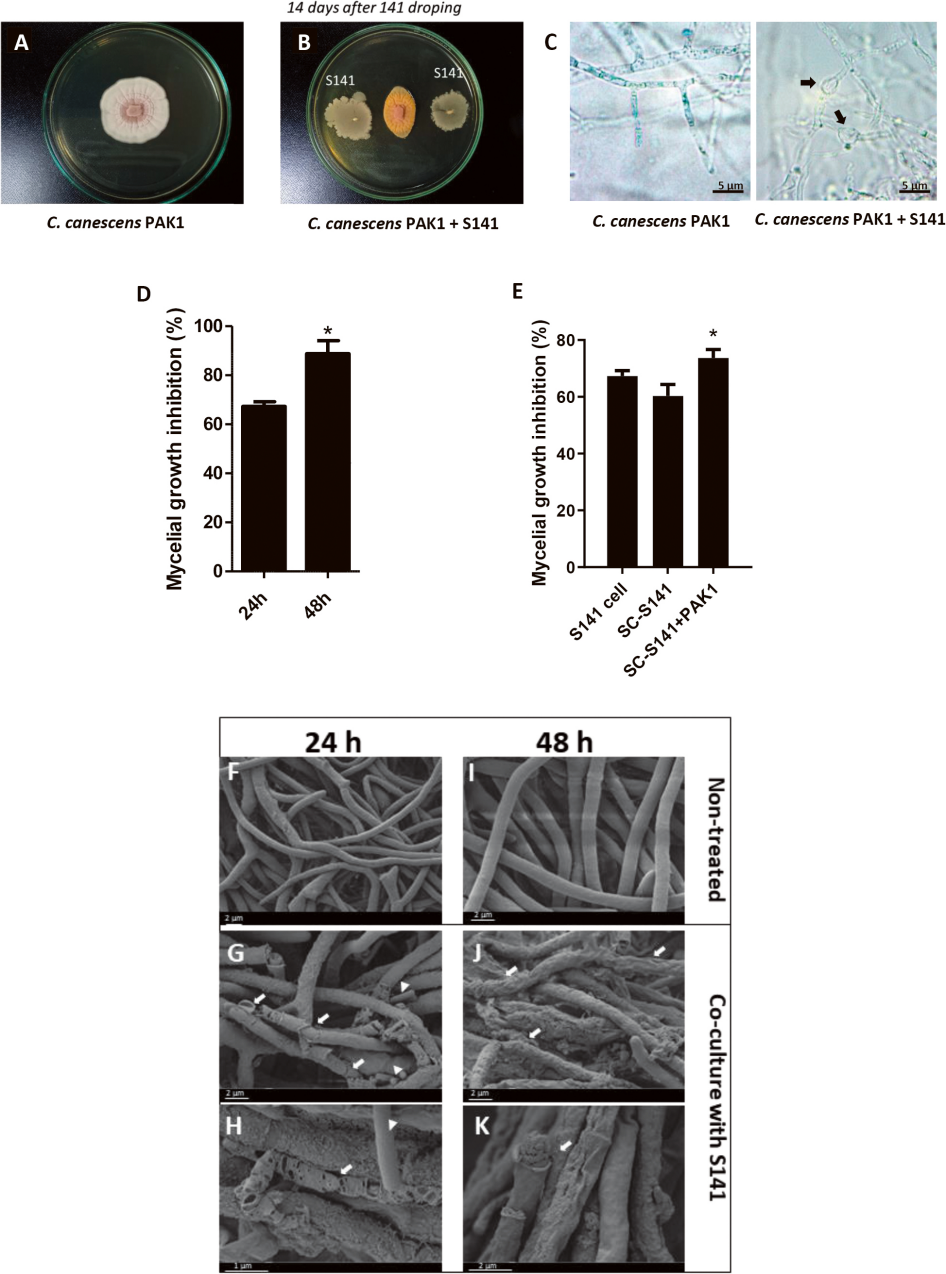
Evaluation of the antifungal activity of *Bacillus velezensis* S141 against *Cercospora canescens* PAK1. The colony morphology of *C. canescens* PAK1 in single and dual cultures on PDA plates (A and B). Hyphal morphology under a light microscope (C) was normal in the single culture of PAK1 and abnormally swollen (black arrows) in the dual culture of PAK1 and S141. Mycelial growth inhibition (%) of *C. canescens* from single and dual cultures with S141 cells 24 and 48‍ ‍h after cultivation (D). Comparison of the mycelial growth inhibition (%) of *C. canescens* by a cultivation in bacterial secretions from the S141 culture (SC-S141) and dual culture of S141+PAK1 (SC-S141+PAK1) with that by bacterial secretions from the dual culture with S141 cells (S141 cells) for 48‍ ‍h (E). SEM observations of *C. canescens* hyphae from the single culture (non-treated) at 24 and 48‍ ‍h (F and I) and damaged hyphae from the coculture with S141 cells at 24 (G and H) and 48‍ ‍h (J and K). White arrows indicate damaged fungal hyphae, while white triangles indicate S141 cells.

The antifungal activity of *B. velezensis* S141 was investigated using the dual culture with *C. canescens* (PAK1) on agar plates and in broth cultures. The results obtained showed the potential of S141 as a biocontrol agent against *C. canescens* growth (Fig. 1). The inhibition of PAK1 on PDA was significantly stronger in the dual culture of PAK1 and S141 versus the single culture of PAK1 (Fig. 1A and B).

Light microscopy of fungal mycelia in the dual culture on agar plates with *B. velezensis* S141 revealed distortions and abnormal swelling that resulted in bulbous structures in hyphal cell walls. These changes were not observed in control mycelia (Fig. 1C). This result was confirmed in the co-culture in liquid medium. S141 inhibited mycelial growth in the upper 60 and 80% within 24 and 48 h, respectively (Fig. 1D). We then investigated whether the above phenomena occurred with the compounds secreted by S141 under normal conditions and with the PAK1 combination. The cell-free secretions of the S141 culture (SC-S141) inhibited mycelial growth to a similar extent as those of the dual culture (S141 cells). The cell-free secretions of S141 and the PAK1 combination significantly increased the rate of inhibition relative to other treatments (Fig. 1E).

PAK1 hyphae from the single and dual cultures with S141 were analyzed at 24 and 48‍ ‍h using SEM to detect morphological changes in *C. canescens* PAK1 hyphae. The images obtained clearly showed the effects of S141 on the morphology of PAK1 mycelia. In the non-treated group, PAK1 mycelia were regular with a smooth cell wall surface (Fig. 1F and I). In contrast, PAK1 hyphae co-cultured with S141 collapsed at 24 and 48‍ ‍h and contained large breakages. Some hyphae showed severe damage, such as shriveling, and distortions, including shedding of the cell wall (Fig. 1G, H, I, J, and K). These results showed that *B. velezensis* S141 exhibited strong antifungal activity against *C. canescens*.

### The potential of B. velezensis S141 as a biocontrol agent for the control of *Cercospora* leaf spot in mungbean

**Fig. 2.**
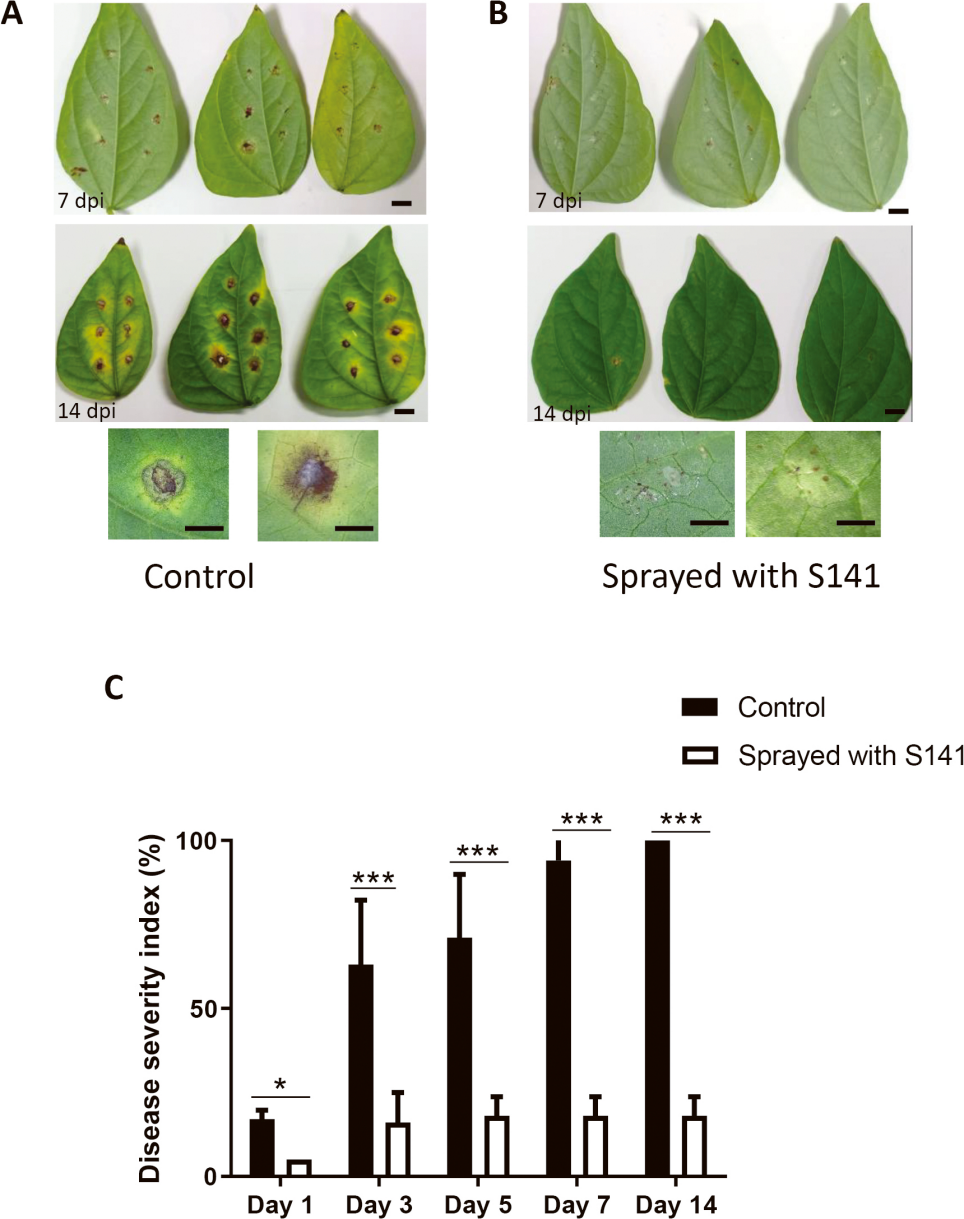
*Cercospora* leaf spot symptoms on mungbean leaves (*Vigna radiata* CN72) infected with *Cercospora canescens* PAK1 7 and 14‍ ‍d post infection (dpi). A shows plants treated with water (Control) and B shows those sprayed with a *Bacillus velezensis* S141 suspension (Sprayed with S141). The disease severity index (%) of mungbean infected with PAK1 1, 3, 5, 7, and 14 dpi after spraying with S141 and the control. Data are the mean±SD of 5 replicates, and the significance of differences was evaluated using a two-way ANOVA (*P<0.05, ***P<0.001). Bars: 1‍ ‍cm

Based on the above results, we examined biocontrol by S141 of *Cercospora* leaf spot in a susceptible mungbean cultivar (*V. radiata* cv. CN72). S141 controlled CLS. Symptoms were significantly weaker in mungbean cultivars treated with S141 than in those treated with the control (Fig. 2A and B). The percentage of disease severity index (%DSI) was significantly reduced (5% to 18%) after the S141 treatment, while that after the control treatment was markedly higher (17% to 100%) (Fig. 2C). These results indicated that S141 exhibited strong antifungal activity against *C. canescens* and significantly reduced the severity of CLS with a control efficiency of 83%. Therefore, *B. velezensis* S141 has potential as a biocontrol agent against *Cercospora* leaf spot disease in mungbean.

### Transcriptomic profile of the biocontrol agent S141 during C. canescens PAK1 inhibition

A comparative transcriptome ana­lysis of S141 from the dual culture with *C. canescens* PAK1 mycelia and the single culture of S141 was conducted to elucidate the mechanisms of action of *B. velezensis* S141 during the inhibition of *C. canescens*. The inhibitory activity of S141 was high at 24‍ ‍h (Fig. 1D), and, thus, the transcriptomic profile of S141 after 24‍ ‍h of PAK1 inhibition was investigated. The total clean reads of the single culture (S141) and double culture (S141+PAK1) were 34.06 and 30.25 Gb, respectively. In total, 33.34 and 29.58 Gb, respectively, were obtained after trimming with an average read length of 150 bp. The Q20 percentages of each library were retained at 97.89 and 97.78%, respectively (Table 1). RNA-seq datasets were deposited in the National Center for Biotechnology Information (NCBI) database under the BioSample accession numbers SAMN31519236 (single culture of S141) and SAMN31519237 (dual culture of S141+PAK1).

**Fig. 3.**
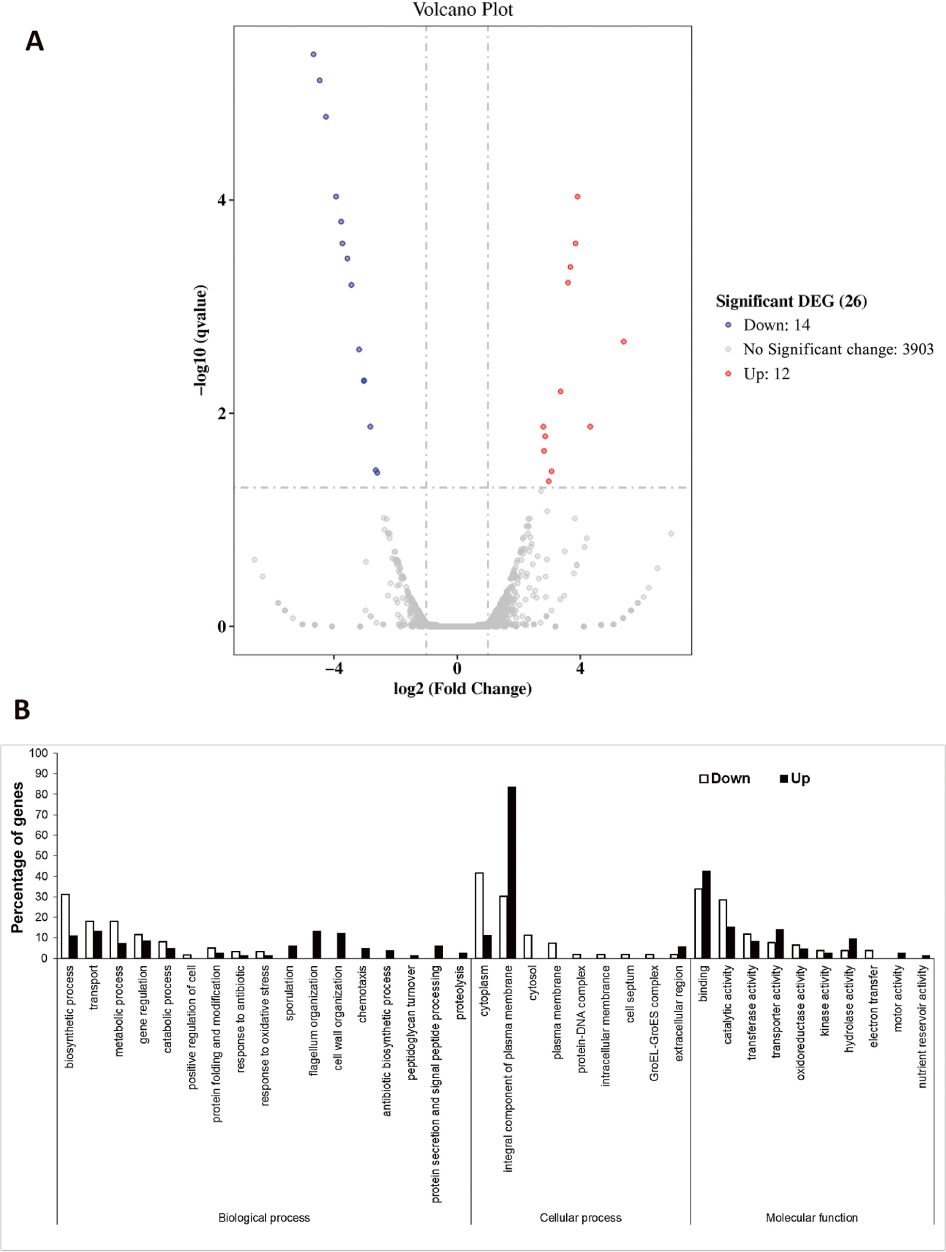
Comparative transcriptomics of *Bacillus velezensis* S141 during *Cercospora canescens* PAK1 inhibition. A volcano plot of the gene expression pattern in strain S141 following its interaction with PAK1 24‍ ‍h post cultivation (A). A purified culture of S141 in PDB was used as the control. The significance of differentially expressed genes (DEG) was defined under the criteria of Padj<0.05 and log2FC >1 or <–1. GO classification of up-regulated (*P*<0.05, log2FC >1) and down-regulated genes (*P*<0.05, log2FC<1) relative to S141 during PAK1 inhibition (B). The X-axis represents GO terms. The Y-axis shows the percentage of genes that were up- and down-regulated.

DEGs between the single culture of S141 and double culture (S141+PAK1) are shown in Fig. 3. Twenty-six DEGs were identified after screening under the criteria of Padj<0.05 and log_2_FC >1 or <–1. There were 12 up-regulated genes and 14 down-regulated genes, while the expression of 3,903 genes remained unchanged (Fig. 3A).

### GO enrichment ana­lysis of DEGs

**Fig. 4.**
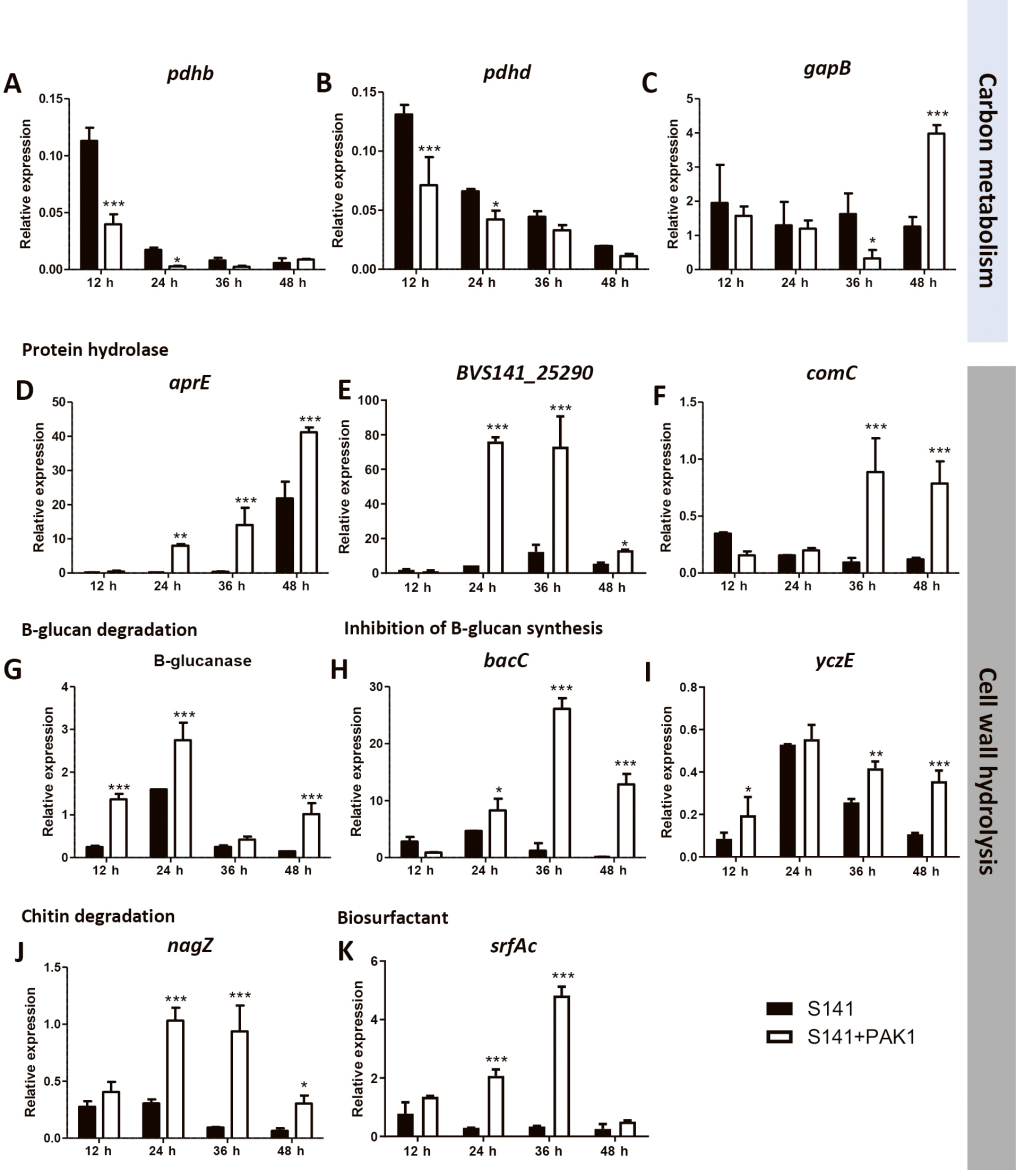
qRT-PCR ana­lysis of data on *Bacillus velezensis* S141 during *Cercospora canescens* PAK1 inhibition. The relative expression of genes involved in carbon metabolism (A, B, and C) and cell wall hydrolysis (D, E, F, G, H, I, J, and K) was observed in a single culture of S141 and compared with that in a dual culture of S141+PAK1 at different time points after cultivation (12, 24, 36, and 48‍ ‍h). Data are the mean±SD of biological triplicates in two independent experiments. Data were analyzed and the significance of differences was evaluated using a two-way ANOVA (*P<0.05; **P<0.01; ***P< 0.001).

To enrich DEGs, genes with *P*<0.05 were considered. In total, 167 up-regulated genes and 114 down-regulated genes were identified and subjected to further ana­lyses. The GO classification of up- and down-regulated genes of *B. velezensis* S141 co-cultivated with *C. canescens* PAK1 facilitated the identification of biological aspects as well as cellular and mole­cular processes (Fig. 4). The results of the biological process classification revealed that the majority of the down-regulated genes were grouped in biosynthetic processes, transport, metabolic processes, gene regulation, and catabolic processes. Only up-regulated genes appeared during the sporulation process, flagellum organization, cell wall organization, chemotaxis, antibiotic biosynthetic process, peptidoglycan turnover, signal peptide processing, protein secretion, and proteolysis. The most diverse genes in the cellular process were detected in the cytoplasm and the integral component of the plasma membrane. Most of the genes in mole­cular function exhibited binding and catabolic activities. The number of up-regulated genes was higher during binding, transport activity, hydrolase activity, motor activity, and nutrient reservoir activity in most pathways (Fig. 3B).

The transcriptome levels of most of the genes involved in carbon metabolism were reduced (Table S2). The expression of genes related to secondary metabolites, antibiotic biosynthesis, and hydrolyses (proteases) was up-regulated (Table S3). Based on these results, 11 representative genes were confirmed by qRT-PCR: three genes involved in carbon metabolism (*pdhB*, *pdhD*, and *gapB*) were from the down-regulation group; six genes related to biocontrol properties (*BVS141_25290*, *aprE*, *bacC*, *nagZ*, *surAc*, and *comC*) were from the up-regulation class; and two genes encode β-glucanase and positive regulator antibiotic production (*yczE*) that correspond to genes with no changes in expression from the NGS.

To elucidate the mechanisms underlying biocontrol by S141 during the inhibition of *C. canescens*, we assessed the relative expression of 11 selected genes at 12-h intervals during cultivation until 48 h. Relative gene expression in Fig. 4 compares expression from the single culture (S141) and dual culture of S141 and PAK1 mycelia (S141+PAK1). The results obtained showed that genes related to the pyruvate dehydrogenase multienzyme complex, including *pdhB* (dihydrolipoamide dehydrogenase) and *pdhD* (pyruvate dehydrogenase E1 component subunit β), were down-regulated at 12 and 24 h. No significant changes were noted in expression levels from 36 to 48‍ ‍h (Fig. 4A and B). The expression of glyceraldehyde-3-phosphate dehydrogenase (*gapB*) did not significantly change from 12 to 24 h. However, *gapB* expression was down-regulated at 36‍ ‍h and up-regulated at 48‍ ‍h (Fig. 4C).

Regarding the gene expression profiles of S141 during PAK1 inhibition, most of the genes involved in cell wall hydrolysis, such as protease-encoding genes (*BVS141_25290* and *aprE*), were significantly up-regulated at 24, 36, and 48‍ ‍h (Fig. 4D and E). The membrane protease *comC* was significantly up-regulated at 36 and 48‍ ‍h (Fig. 4F). The expression of β-glucanase and the positive regulator for antibiotic production (*yczE*) showed early induction at 12‍ ‍h (Fig. 4G). Moreover, the gene expression of biosurfactant (*srfAc*), the antibiotic bacilysin (*bacC*), and N-acetyl glucosaminidase (*nagZ*) was up-regulated from 24‍ ‍h (Fig. 4K, H, and I). Based on the results from the transcriptome ana­lysis (NGS) and qRT-PCR, relative expression at 24‍ ‍h from both observations was mostly consistent, except for *gapB*, β-glucanase, and positive regulator *yczE* genes (Fig. S2). The expression of *gapB* from NGS was down-regulated, while qRT-PCR showed the down-regulation of this gene at 36‍ ‍h (Fig. S2C). The differential expression of β-glucanase and *yczE* genes was not detected by NGS; however, qRT-PCR data showed significant up-regulation from 12‍ ‍h (Fig. S2G and I). This may be attributed to uncontrollable factors in biological experiments. Collectively, these results demonstrate the potential of *B. velezensis* S141 as a biocontrol agent for *C. canescens* PAK1 through the processing of gene regulatory networks for antifungal mechanisms, including protein hydrolysis, the degradation and inhibition of β-glucan, chitin degradation, and the formation of biosurfactants and biofilms.

## Discussion

*Cercospora* leaf spot disease caused by *C. canescens* is a serious disease in mungbean (*V. radiata*). We herein report the biocontrol properties of *B. velezensis* S141, which was previously identified as an effective PGPR for soybean (*Glycine max* [L.] Merr.) after a co-inoculation with *B. diazoefficiens* USDA110 (Sibponkrung *et al.*, 2020). S141 efficiently inhibited the mycelial growth of *C. canescens* PAK1 (Fig. 1D) and damaged the hyphal structure (Fig. 1C, G, H, I, J, and K). The biocontrol properties of S141 were also investigated with mungbean (*V. radiata*) challenged with *Cercospora* leaf spot disease. The results obtained confirmed the potential of *B. velezensis* S141 as a biocontrol agent against *Cercospora* leaf spot disease in mungbean.

The genus *Bacillus* has recently been recognized as a promising biological control agent that has beneficial effects on plant promotion (Borriss, 2011; Chen *et al.*, 2020). For example, *B. velezensis* F21 and *B. amyloliquefaciens* GKT04 exerted biocontrol against *Fusarium* wilt (Jiang *et al.*, 2019; Tian *et al.*, 2021). *B. velezensis* strain E2 exhibited antifungal activity against *Aspergillus flavus* (Li *et al.*, 2022), and strain MRP4490 inhibited soil-borne plant pathogens, including *Macrophomina phaseolina*, *Rhizoctonia solani*, *Sclerotinia sclerotiorum*, and *Botrytis cinerea* (Teixeira *et al.*, 2021). These strains all contain gene clusters that encode secondary metabolites and hydrolytic enzymes, which may be antagonistic against pathogen fungi. However, the biocontrol properties of different strains may involve different mechanisms.

Cell lysate and hyphal irregularities observed after the S141 treatment may be attributed to the activities of hydrolytic enzymes that are present in the genome of the genus *Bacillus*. The majority of biocontrol agents are secreted into environmental cultures, including proteases and hydrolase enzymes for cell wall degradation and antibiotic agents (Shi *et al.*, 2010; Ji *et al.*, 2020; Rong *et al.*, 2020). As expected, bacterial secretions from the single culture of S141 and dual culture of S141 and PAK1 mycelia showed potential as biocontrol agents to inhibit mycelial growth, similar to the direct treatment of S141 cells. Bacterial secretions from S141+PAK1 enhanced antifungal activity (Fig. 1E). In contrast, secretions from PAK1 (SC-PAK1) did not affect mycelial growth differently from the control (PAK1 cultured in PDB) (Fig. S1). This result suggests that the biological mechanisms underlying the antifungal activity of S141 were enhanced by PAK1 mycelia during cultivation. This hypothesis is reasonable because *N*-acetylglucosamine (GlcNAc; a monomer of chitin) accumulated in the medium during *C. canescens* inhibition.

**Fig. 5.**
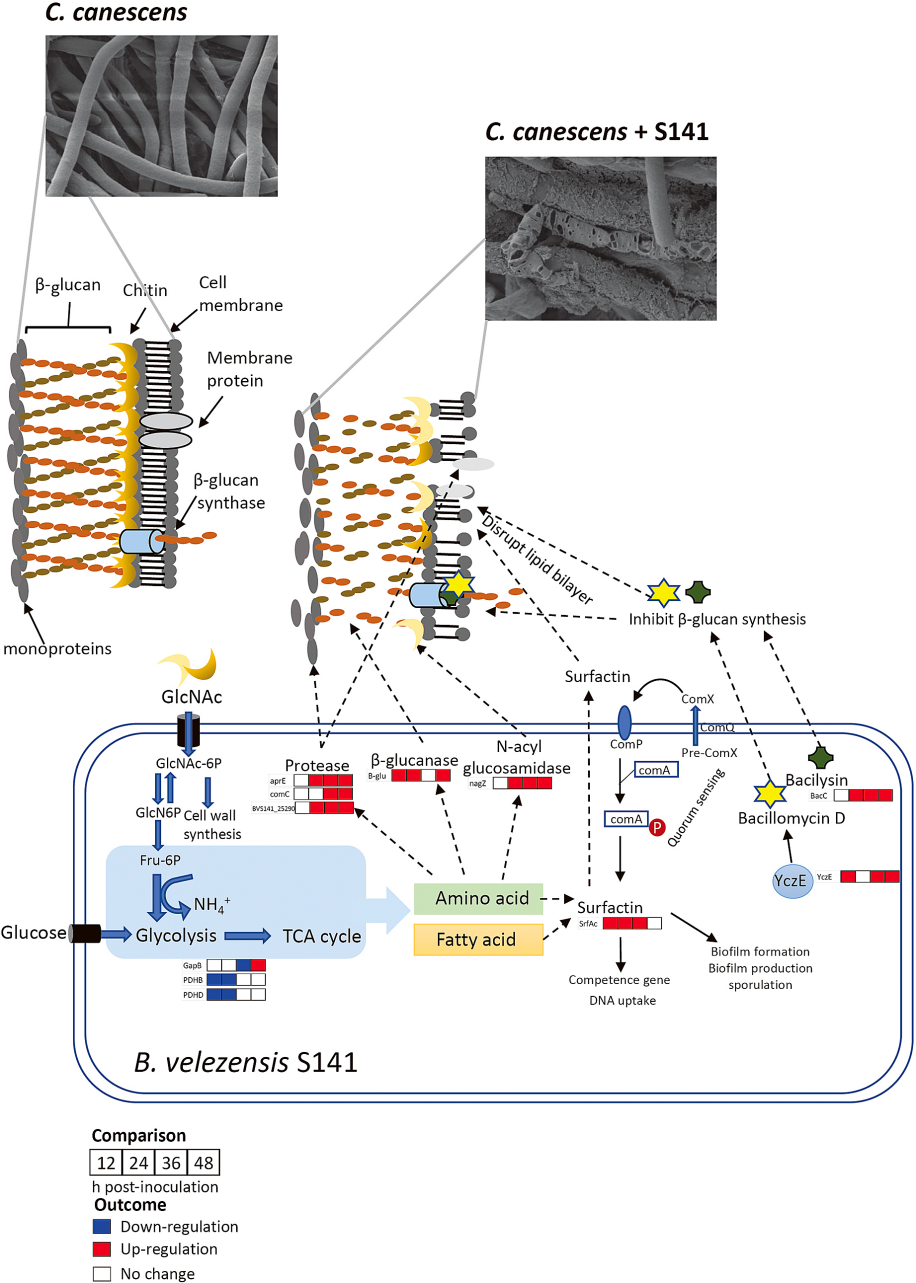
Schematic overview of the antifungal activity of *Bacillus velezensis* S141 against *Cercospora canescens*. The putative mechanisms were predicted based on the results of the qRT-PCR ana­lysis at different time points. The mechanisms underlying fungal hydrolysis by S141 during the *C. canescens* interaction were proceeded by enzymatic hydrolases, including protease, β-glucanase, and N-acyl glucosamidase, acting on membrane proteins, β-glucan, and chitin. Secondary metabolites, including surfactin, bacilysin, and bacillomycin D, were activated to disrupt the lipid bilayer and inhibit cell wall biosynthesis. Carbon metabolism in S141 was also suppressed during *C. canescens* inhibition. SEM images are shown in Fig. 1.

Previous studies suggested that the accumulation of N-acetylglucosamine (NAG) during the autolytic degradation of vegetative mycelia triggered the development of and antibiotic production by *Streptomyces* species (Rigali *et al.*, 2008; Świątek *et al.*, 2012). Moreover, GlcNAc is an important source of nutrients for both catabolic and anabolic purposes in the genus *Bacillus*. The uptake of GlcNAc may support cell wall biosynthesis or alternatively serve as a favorable nutrient source for major biological processes, such as glycolysis, the tricarboxylic acid (TCA) cycle, respiration, nucleic acids, nitrogen, and fatty acid metabolism (Niu *et al.*, 2020). The utilization of GlcNAc from S141 during PAK1 antagonism may provide a nutrient source for metabolism. However, the present results showed that the genes involved in central carbon metabolism were down-regulated (*i.e.*, *pdhB*, *pdhD*, and *gapB*) (Table S2 and Fig. 5). This result is consistent with previous findings on the limits of secondary metabolites from carbon sources in cultures. Secondary metabolites are produced in the stationary phase, which has low nutrients and a low growth rate. The production of some antibiotics was previously shown to be affected by glucose and other carbohydrates (Sánchez *et al.*, 2010; Demain, 2020). Therefore, the down-regulation of genes involved in central carbon metabolism may be regulated by systematic insight during secondary metabolism.

The main functions of antifungal properties are commonly considered to be in the production of hydrolytic enzymes. The actions of these enzymes markedly affect the fungal cell wall structure by degrading membrane proteins, the protective layer of chitin, glucans (β-1,3- and β-1,6-glucans), and mannoproteins on the outer surface. Chitin is‍ ‍composed of a linear polymer of β-1,4-linked N-acetylglucosamine (NAG) residues. There are two main types of enzymes that degrade chitin: endochitinases (1,4-β-poly-N-acetylglucosaminidases) and exochitinases (β-N-acetyl-hexosaminidases encoded by *nagZ*). Chitin degradation releases NAG 1,3 dimers and monomers (Horsch *et al.*, 1997). The results of the transcriptome ana­lysis (NGS) and qRT-PCR showing differential expression revealed that *nagZ* expression in *B. velezensis* S141 was significantly up-regulated at 24, 36, and 48‍ ‍h using an antagonist against *C. canescens* PAK1. Moreover, the relative expression of β-glucanase was significantly up-regulated by S141+PAK1 at 12, 24, and 48‍ ‍h (Fig. 4G). These results indicate that the antifungal activity of S141 was initiated by glucan-chitin degradation. This, in turn, resulted in extensive thinning and a less dense hyphal network than in the mycelia of the non-treated fungus. Besides glucan-chitin-degrading enzymes, some antibiotics, *e.g.*, bacilysin encoded by the *bacABCDE* operon and bacillomycin D encoded by the *bamABC* operon, inhibit fungal growth by blocking the function of B-glucosamine synthase, leading to the rapid breakdown of the cell wall and increased cell permeability (Mariappan *et al.*, 2012; Lin *et al.*, 2022). In the present study, S141 after 24‍ ‍h of PAK1 inhibition showed the significantly up-regulated expression of *bacC* genes, which are required for the biosynthesis of bacilysin. Moreover, the expression of genes encoding the positive regulator *yczE* (essential for bacillomycin D synthesis) (Koumoutsi *et al.*, 2007) was significantly up-regulated during PAK1 inhibition (Fig. 4H). Previous studies reported that bacillomycin D inhibited the synthesis of β-1,3-glucan and disrupted the lipid bilayer, thereby affecting cell membrane permeability and, ultimately, leading to cell death (Wu *et al.*, 2020; Lin *et al.*, 2021).

Proteolytic enzymes also play a role in biocontrol against fungal pathogens by hydrolyzing the peptide bonds of membrane proteins, thereby causing cell lysis and subsequent cell death. For example, the proteases produced by *Trichoderma harzianum* and *Pseudomonas aeruginosa* M-1001 are important for the biological control of fungal pathogens (Tseng *et al.*, 2008; Zhang *et al.*, 2012). In the present study, the cocultivation of *B. velezensis* S141 with *C. canescens* PAK1 induced the expression of genes for proteolytic activity, including serine alkaline protease (*aprE*), protease (*BVS141_25290*), and membrane protease (*comC*). Differential expression was detected from 24 to 48‍ ‍h in the dual culture (Fig. 4D, E, and F).

Serine protease antagonism against fungi has been reported in *B. licheniformis* W10, which plays a role in *B. cinerea* inhibition (Ji *et al.*, 2020). *Aureobasidium pullulans* PL5 has been shown to secrete alkaline extracellular serine proteases, which contribute to biocontrol activities against some postharvest pathogens of apple and peach (*Monilinia laxa*, *B. cinerea*, and *Penicillium expansum*; Zhang *et al.*, 2012). These findings indicate that some of the protease-encoded genes were induced through communication between *B. velezensis* S141 and *C. canescens* PAK1. Although S141 contains multiple copies of protease genes in the genome, the differential expression of these genes was not detected (Table S4).

Besides the hydrolytic enzymes described above, *Bacillus* strains also secrete potent biosurfactants, including surfactin. Surfactin is a cyclic lipopeptide that comprises a peptide loop attached to a hydrophobic fatty acid chain encoded by the *srfA* operon comprising four open reading frames: *srfAA*, *srfAB*, *srfAC*, and *srfAD* (Rahman *et al.*, 2021). Surfactin has been shown to efficiently decrease the surface tension of water, thereby causing cells to lyse through the disruption of the phospholipid bilayer (Harwood *et al.*, 2018). Previous studies revealed that the antagonistic activity of *Bacillus* sp. FJAT-14262 against *Fusarium oxysporum* was attributed to the secreted lipopeptide surfactin (Chen *et al.*, 2017). In the present study, the differential expression of *srfAC* in S141 during PAK1 inhibition was detected from 12 to 36‍ ‍h post-cultivation (Fig. 4K). These results indicate that S141 exhibits strong antagonistic activity due to cell wall hydrolysis and the inhibition of cell wall biosynthesis. S141 also disrupted the structure of the lipid bilayer via the lipopeptide surfactin (Fig. 5).

The synthesis of surfactin is regulated by a quorum sensing system encoded by the *comQXPA* gene cluster. The secretion of modified ComX via the ComQ system leads to the accumulation of ComX outside cells. Upon reaching a critical cell density at the stationary phase, ComX interacts with the N-terminal sensory domain of ComP to phosphorylate the transcriptional activator ComA (ComA-P). ComA-P directly binds to the promoter region of the *srfA* operon and initiates the transcription of the adjacent gene. At this stage, cells stimulate sporulation and the formation of biofilms. ComA-P may also enter competent cells and lead to process DNA uptake from the extracellular matrix (Rahman *et al.*, 2021). Moreover, the accumulation of surfactin in media with high cell concentrations may also promote cellular motility due to reduced surface tension between cells and a substrate (Ghelardi *et al.*, 2012). GO is classified as a biological process, and the genes involved in sporulation and flagella organization are only present in the class of up-regulated genes (Fig. 3B). This result indicates that *B. velezensis* S141 in the stationary phase (at 24‍ ‍h) performed quorum sensing and induced the synthesis and secretion of surfactin. The accumulation of surfactin in the substrate may have promoted sporulation, biofilm production, flagellar development, and cellular motility (Fig. 5).

The secondary metabolites by *B. velezensis* S141 during antifungal activity were examined. The results of the transcriptome ana­lysis revealed that S141 during cultivation with or without PAK1 mycelia expressed the genes required for the biosynthesis of other antifungal polyketides (PKs) and non-ribosomal peptides (NRPS). There were three gene clusters that encoded for the biosynthesis of PKs: bacillaene (*beaSII*), macrolactin (*mlnABCDEFGHI*), and difficidin (*dnfBCFGHIJKMXY*). Regarding the biosynthesis of NRP, other genes responsible for bacillibactin, bacillomycin D, bacilysin, and surfactin were detected; however, the expression of these genes did not change between treatments (Table S4). Collectively, these results suggest the significant potential of *B. velezensis* S141 as a biocontrol agent against *C. canescens* fungi due to its ability to produce a large collection of cell wall hydrolytic enzymes and several effective secondary metabolites.

The biocontrol effects exerted by *B. velezensis* S141 and other antagonistic *Bacilli* may proceed via different mechanisms. Besides the direct effects of hydrolytic enzymes and the spectrum of secreted secondary metabolites, the stimulation of induced systemic resistance (ISR) is of importance. ISR in plants is triggered by a range of secondary metabolites leading to an induced plant defense reaction through the stimulation of different signaling pathways, such as jasmonic acid (JA), ethylene (ET), and salicylic acid (SA). Biocontrol by *B. velezensis* FZB42 revealed that the production of surfactin from this strain induced JA/ET-dependent ISR when challenged with a plant pathogen (Chowdhury *et al.*, 2015). The direct treatment of crude surfactin also suppressed disease by inducing plant defense factors, such as hydrogen peroxide (H_2_O_2_) and phenolic compounds (Rahman *et al.*, 2015). In the present study, the biocontrol potential of *B. velezensis* S141 against *Cercospora* leaf spot on mungbean was facilitated by the induction of plant systemic resistance in addition to direct antibiosis by a spectrum of secreted compounds.

## Conclusion

*B. velezensis* S141 exhibited antifungal activity against *C. canescens* PAK1 on plate and liquid cultures. Cell-free secretions from single cultures of S141 and dual cultures of S141+PAK1 exhibited antifungal activity. However, cell-free secretions from the S141+PAK1 culture enhanced fungal inhibition. These results suggest that the antifungal activity of S141 promotes interactions with fungal pathogens. Through the combination of transcriptomic sequence data and qRT-PCR derived from strain S141 during PAK1 inhibition, we herein demonstrated that the biosynthesis of cell wall hydrolase enzymes was enhanced during *C. canescens* inhibition by *B. velezensis* S141. Examples include protease, β-glucanase, and N-acyl glucosaminase as well as secondary metabolites, such as surfactin, bacilysin, and bacillomycin D. Carbon metabolism was, in turn, down-regulated. Other biological functions, such as quorum sensing, sporulation, biofilm production, flagellar development, and cellular motility, may be involved in the biosynthesis of these antifungal metabolites. These results provide a comprehensive insight into the biological functions underlying the antifungal activity of *B. velezensis* S141 against *C. canescens*.

## Citation

Songwattana, P., Boonchuen, P., Piromyou, P., Wongdee, J., Greetatorn, T., Inthaisong, S., et al. (2023) Insights into Antifungal Mechanisms of *Bacillus velezensis* S141 against *Cercospora* Leaf Spot in Mungbean (*V. radiata*). *Microbes Environ ***38**: ME22079.

https://doi.org/10.1264/jsme2.ME22079

## Supplementary Material

Supplementary Material

## Figures and Tables

**Table 1.** Summary of read numbers, average read length, number of reads after trimming, and percentage retained of raw reads based on RNA-seq data from a single culture of *Bacillus velezensis* S141 and double culture of S141 combined with *Cercospora canescens* PAK1 (S141+PAK1)

	**S141**	**S141+PAK1**
No. of reads	34,059,754	30,250,556
Average read length (bp)	150	150
Number of reads after trimming	33,341,093	29,578,993
Percentage retained	97.89%	97.78%
