# Supplementary material for: Insights into Antifungal Mechanisms of Bacillus velezensis S141 against Cercospora Leaf Spot in Mungbean (V. radiata)
Source: Microbes Environ. 2023 Mar 18;38(1):ME22079. doi: 10.1264/jsme2.ME22079 (PMC10037098; doi:10.1264/jsme2.ME22079)
Supplement: Supplementary file 1 — Supplementary Material [file 38_22079_s1.pdf]

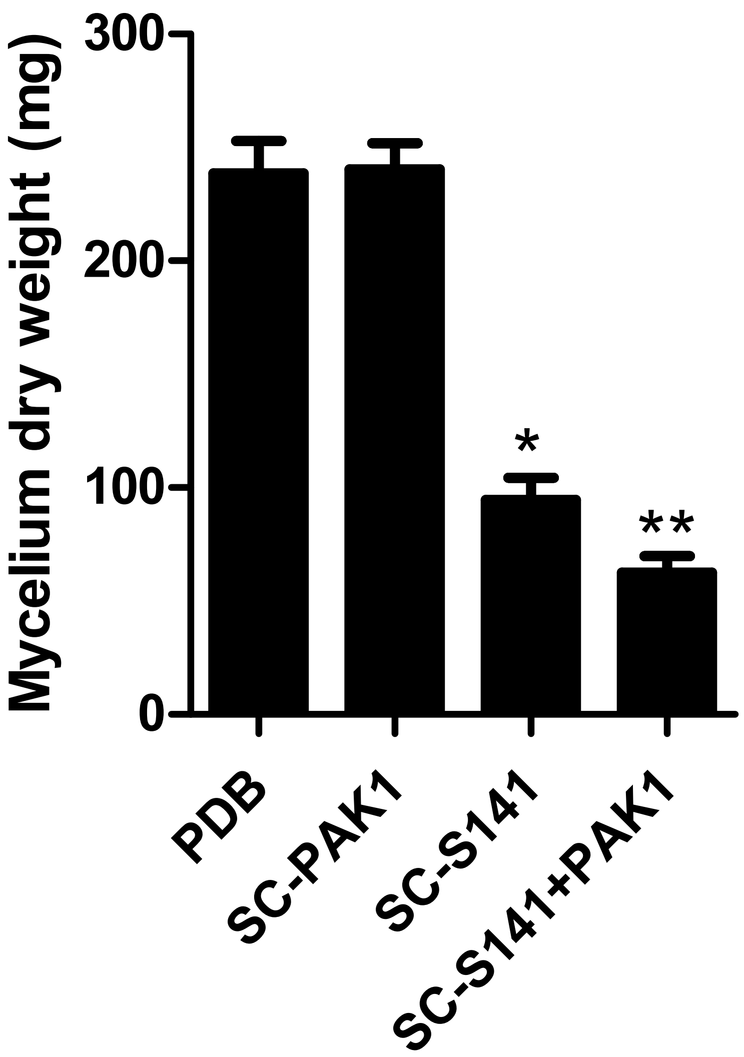
Mycelium dry weight (mg)
300
200
100
0
*
**
PDB
SC-PAK1
SC-S141
SC-S141+PAK1

Figure S1

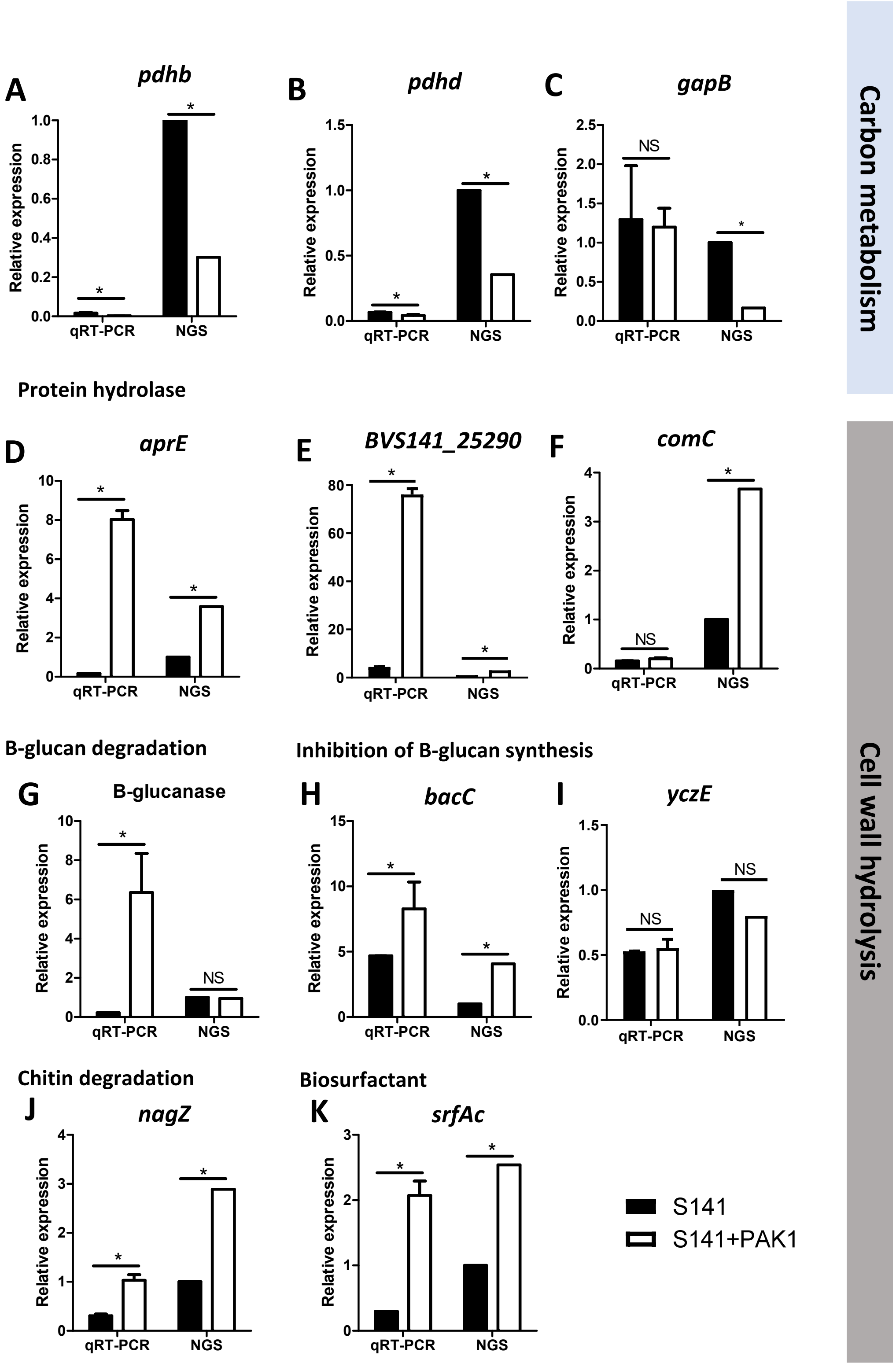

Carbon metabolism
Cell wall hydrolysis
A
pdhb
B
pdhd
C
gapB
Protein hydrolase
D
aprE
E
BVS141_25290
F
comC
B-glucan degradation
Inhibition of B-glucan synthesis
G
B-glucanase
H
bacC
I
yczE
Chitin degradation
Biosurfactant
J
nagZ
K
srfAc
Relative expression
qRT-PCR
NGS
NS
S141
S141+PAK1

Figure S2

Table S1 List of primers used in this study

Gene name	Primer name	Primer sequence (5'-3')
Glyceraldehyde-3-phosphate dehydrogenase	gapB.F	GGCCTGATGACGACTGTTCA
	gapB.R	CGTTTTTGCGGCTCTTTGGA
Pyruvate dehydrogenase E1 component subunit beta	pdhB.F	ATCATCACATACGGCGCGAT
	pdhB.R	TGAGCTTCCTGCACGACAAT
Dihydrolipoamide dehydrogenase	pdhD.F	CTGAACTGGCTTCTGTCGGT
	pdhD.R	ATGTCGGGTGAGCGTGAATT
Pyruvate dehydrogenase E1 subunit alpha	Pry-DH.F	CCTTCCGGGTTATCGAGACGT
	Pry-DH.R	AGCTACACCTGCGGTTTGAA
Biosurfactant SrfAC protein	SrfAC.F	TTGAGGAACAGGCAAGCGAT
	SrfAC.R	CCGTGCCGCATTCAGTATTG
Protease	protease.F	TTTTGCAGTGATCGTGTCGG
	protease.R	GACTAATGCCAGGGTCACGA
Serine protease	aprE.F	AACGGAATTGAGTGGGCGAT
	aprE.R	TGGTGCCATGACATCAAGCT
Membrane protease and transmethylase	comC.F	GCGCAGGTGATGTGAAACTG
	comC.R	GCAAACGGAAATGGCTGCTT
Bacilysin biosynthesis BacD protein	bacC.F	GCCCGACATTCCCGCATATA
	bacC.R	AGCGTGCCTTCGTTGTTTTC
Beta-glucanase	B-Glu.F	GTGCTGCTGCTTCTTGTCAC
	B-Glu.R	TCCCCGCAGTCAAACTTGTT
N-acetyl glucosaminidase lipoprotein (NagZ)	nagZ.F	GCTTTAACGGCGTCATCGTC
	nagZ.R	CGGTTTTCTGTTTGCAGCGA
16S rRNA	PBA338.F	ACTCCTACGGGAGGCAGCAG
	PRUN518.R	ATTACCGCGGCTGCTGG

Table S2. List of Differentially Expressed Genes (DEGs) from down-regulated genes at P-value<0.05 and $log_2FC<1$

	Gene Name	Gene ID	Description	Log_2FC	P-value
**Biosynthetic process**	*hisZ*	BVS141_33320	ATP phosphoribosyl transferase regulatory subunit	-2.2412	1.45E-03
	hisF	BVS141_33260	imidazole glycerol phosphate synthase subunit HisF	-1.5932	2.14E-02
	hisA	BVS141_33270	1-(5-phosphoribosyl)-5-[(5-phosphoribosylamino) methylidene amino] imidazole-4-carboxamide isomerase	-1.9579	5.19E-03
	hisD	BVS141_33300	bifunctional histidinal dehydrogenase/histidinol dehydrogenase	-1.4949	2.76E-02
	hisB	BVS141_33290	imidazole glycerol-phosphate dehydratase	-1.4751	3.47E-02
	hisI	BVS141_33250	phosphoribosyl-AMP cyclohydrolase	-1.8625	7.60E-03
	BVS141_16060	BVS141_16060	dihydroorotate dehydrogenase	-3.0254	1.88E-05
	pyrF	BVS141_16070	orotidine 5'-phosphate decarboxylase	-3.0296	2.03E-05
	pyrB	BVS141_16010	aspartate carbamoyl transferase	-4.2601	1.26E-08
	BVS141_16030	BVS141_16030	carbamoyl phosphate synthase small subunit	-4.4647	3.83E-09
	BVS141_00900	BVS141_00900	cysteine synthase	-1.4363	3.12E-02
	hisH	BVS141_33280	imidazole glycerol phosphate synthase subunit HisH	-1.4222	3.94E-02
	BVS141_16020	BVS141_16020	dihydroorotase	-4.6636	1.09E-09
	pyrK	BVS141_16050	dihydroorotate dehydrogenase electron transfer subunit	-3.1849	8.98E-06
	pyrAB	BVS141_16040	carbamoyl phosphate synthase large subunit	-3.7679	2.43E-07
	pyrE	BVS141_16080	orotate phosphoribosyl transferase	-3.7243	5.20E-07
	BVS141_38620	BVS141_38620	adenylosuccinate synthetase	-1.3765	3.86E-02
	hemY	BVS141_10670	protoporphyrinogen oxidase	-1.3828	3.79E-02
	gsaB	BVS141_09000	glutamate-1-semialdehyde aminotransferase	-1.3637	4.06E-02
**Transport**	*BVS141_03820*	BVS141_03820	Membrane-bound lytic murein transglycosylase F	-1.4275	3.29E-02
	yckJ	BVS141_03810	Inner membrane amino-acid ABC transporter permease YecS	-1.3225	4.77E-02
	RBSB	BVS141_34340	ribose ABC transporter%2C ribose-binding lipoprotein	-1.5099	4.95E-02
	mccB	BVS141_25190	cystathionine beta-lyase	-1.3105	4.93E-02
	sacP	BVS141_36460	SacP protein	-2.3582	1.17E-03
	treP	BVS141_08190	TreP protein	-2.1621	1.20E-02
	ywoD	BVS141_34850	major facilitator superfamily protein YwoD	-1.7645	1.04E-02

	ywkB	BVS141_35400	Auxin efflux carrier family protein	-1.9583	4.13E-03
	BVS141_31090	BVS141_31090	MFS transporter DHA2 family multidrug resistance protein B	-1.3639	4.20E-02
	atpI	BVS141_35240	ATP synthase subunit I	-1.6098	1.67E-02
	fruA	BVS141_14840	phosphotransferase system fructose-specific enzyme IIABC component	-2.2916	8.26E-04
**Metabolic process**	*nagA*	BVS141_33380	N-acetyl glucosamine-6-phosphate deacetylase	-1.5886	1.77E-02
	gabD	BVS141_04180	succinate-semialdehyde dehydrogenase	-1.4870	2.59E-02
	gapB	BVS141_26990	glyceraldehyde-3-phosphate dehydrogenase	-2.5946	2.30E-04
	BVS141_35410	BVS141_35410	malate dehydrogenase	-1.6028	1.70E-02
	ytcI	BVS141_27450	acetyl-CoA synthetase	-1.3407	4.40E-02
	upp	BVS141_35250	uracil phosphoribosyl transferase	-1.3706	4.63E-02
	pdhB	BVS141_15130	pyruvate dehydrogenase E1 component subunit beta	-1.7305	1.00E-02
	BVS141_15120	BVS141_15120	pyruvate dehydrogenase E1 subunit alpha	-1.3938	3.64E-02
	pdhD	BVS141_15150	dihydrolipoamide dehydrogenase	-1.4938	2.52E-02
	yjiC	BVS141_12680	glycosyltransferase%2C possible macrolideglycosyltransferase	-2.2421	1.29E-03
	yngF	BVS141_19100	enoyl-CoA hydratase	-5.8109	3.48E-02
**Gene regulation**	*BVS141_27370*	BVS141_27370	site-specific DNA-methyltransferase	-1.3110	4.89E-02
	BVS141_15990	BVS141_15990	bifunctional pyrimidine regulatory protein PyrR uracil phosphoribosyl transferase	-2.0180	3.03E-03
	ctsR	BVS141_01180	transcriptional regulator CtsR	-1.3803	3.86E-02
	trmU	BVS141_25460	tRNA-specific 2-thiouridylase MnmA	-1.3376	4.44E-02
	yhjN	BVS141_11060	integral inner membrane protein	-1.4141	3.50E-02
	BVS141_32050	BVS141_32050	HTH-type transcriptional regulator	-1.9099	4.72E-03
	nagR	BVS141_33400	transcriptional regulator GntR family	-1.8554	5.95E-03
**catabolisum process**	*yjiB*	BVS141_12670	Cytochrome P450	-2.0254	4.04E-03
	putM	BVS141_31050	proline dehydrogenase 1	-1.7888	8.32E-03
	iolE	BVS141_37950	2-keto-myo-inositol dehydratase	-2.2491	1.96E-02
	acuC	BVS141_27830	Acetoin utilization protein AcuC	-1.4840	2.75E-02
	abnA1	BVS141_26800	arabinan endo-1%2C 5-alpha-L-arabinosidase AbnA	-1.4980	4.34E-02
**Positive regulation of cell**	*spoIIM*	*BVS141_22480*	Stage II sporulation protein M	-1.4682	3.14E-02

**Protein folding and modification**	*hslO*	BVS141_00880	33 kDa chaperonin	-1.3399	4.46E-02
	groL	BVS141_06670	60 kDa chaperonin Protein Cpn60	-1.5348	2.16E-02
	sufA	BVS141_30370	chaperone involved in Fe-S cluster assembly	-1.3731	3.99E-02
**Response to antibiotic**	*BVS141_11070*	BVS141_11070	Quinolone resistance protein NorA	-1.9227	4.48E-03
	BVS141_06110	BVS141_06110	methylenomycin A resistance protein	-1.6249	2.80E-02
**Response to oxidative stress**	*trxB*	BVS141_33190	thioredoxin-disulfide reductase	-1.8481	6.15E-03
	yoxD	BVS141_19430	3-ketoacyl-ACP reductase	-1.4035	3.66E-02
**Others**	*BVS141_06050*	BVS141_06050	ISLmo1-like transposase	-6.3178	9.22E-03
	BVS141_27700	BVS141_27700	hypothetical protein	-5.8109	3.48E-02
	BVS141_36480	BVS141_36480	YwcI protein	-5.8109	3.48E-02
	BVS141_12230	BVS141_12230	ABC transporter ATP-binding protein	-3.9331	1.00E-07
	BVS141_12220	BVS141_12220	multidrug ABC transporter permease	-3.4346	1.91E-06
	ypuF	BVS141_22200	SMC interacting protein/Metal-dependent hydrolase%2C possible protease	-2.0348	3.05E-03
	BVS141_32950	BVS141_32950	galactoside permease	-1.9867	2.75E-02
	ykuO	BVS141_14630	BNR repeat-containing protein	-1.9467	1.29E-02
	yclG	BVS141_03890	Putative uronase	-1.9363	3.23E-02
	nrdI	BVS141_17900	ribonucleotide reductase stimulatory protein	-1.8985	5.26E-03
	BVS141_12240	BVS141_12240	AbrB family transcriptional regulator	-1.8687	1.25E-02
	yqkC	BVS141_22620	Uncharacterised protein family YqkC	-1.7914	9.07E-03
	ykvR	BVS141_14230	Uncharacterized protein YkvR	-1.7800	2.39E-02
	BVS141_18280	BVS141_18280	Uncharacterized protein	-1.6363	3.53E-02
	ymzA	BVS141_17890	Uncharacterized protein YmzA	-1.5897	2.02E-02
	ykzT	BVS141_14480	Uncharacterized protein YkzT	-1.5538	2.03E-02
	BVS141_11480	BVS141_11480	DNA methyltransferase	-1.5387	2.17E-02
	BVS141_30740	BVS141_30740	HTH transcription regulator	-1.5196	2.30E-02
	BVS141_15490	BVS141_15490	YlbB protein	-1.5176	2.53E-02
	BVS141_11080	BVS141_11080	HTH-type transcriptional regulator	-1.4969	2.61E-02

ypdA	BVS141_21930	thioredoxin reductase	-1.4684	2.78E-02
ypaA	BVS141_38910	Predicted membrane protein YpaA	-1.4636	3.36E-02
ymaD	BVS141_17810	Organic hydroperoxide resistance protein	-1.4634	3.02E-02
BVS141_29650	BVS141_29650	kinase-associated protein B	-1.4513	4.40E-02
yhbJ	BVS141_09570	multidrug resistance protein A	-1.5939	1.93E-02
yoaK	BVS141_17760	UPF0700 transmembrane protein YoaK	-1.4931	2.71E-02
BVS141_03720	BVS141_03720	HTH-type transcriptional activator HxlR	-2.9631	4.73E-03
yybR	BVS141_38790	transcriptional regulator	-2.3844	7.11E-04
ykvN	BVS141_14200	HTH-type transcriptional regulator YkvN	-2.1063	3.74E-03
ycnC	BVS141_04120	HTH-type transcriptional regulator YcnC	-1.8245	1.05E-02
yhbI	BVS141_09560	HTH-type transcriptional regulator Hpr	-2.1959	1.70E-03
fruR	BVS141_14820	FruR protein	-1.7795	8.53E-03
BVS141_26810	BVS141_26810	peptidase M28	-1.7565	9.11E-03
fruK	BVS141_14830	1-phospho fructokinase	-2.8212	6.12E-05
ywoC	BVS141_34860	isochorismatase hydrolase	-1.9807	4.68E-03
yjcK	BVS141_30350	ribosomal-protein-alanine N-acetyltransferase	-6.5778	4.17E-03
azoR2	BVS141_31820	azoreductase	-1.9770	3.95E-03
yqiG	BVS141_23350	NADH-dependent flavin oxidoreductase	-1.6528	1.38E-02
BVS141_16000	BVS141_16000	uracil permease	-3.5644	8.11E-07
yvrD	BVS141_31400	short-chain dehydrogenase/reductase SDR	-1.8988	6.63E-03
BVS141_06440	BVS141_06440	quinone oxidoreductase	-1.3232	4.91E-02
BVS141_36500	BVS141_36500	luciferase family protein	-2.1917	1.37E-03
yxeI	BVS141_37110	choloylglycine hydrolase	-2.6456	2.01E-04
yvcI	BVS141_33180	NUDIX hydrolase	-1.3416	4.43E-02
ytcJ	BVS141_27440	metal-dependent hydrolase	-1.3965	3.61E-02
bshC	BVS141_15670	malate glucosamine cysteine ligase	-1.3991	3.61E-02
BVS141_09580	BVS141_09580	drug resistance MFS transporter	-1.3901	3.88E-02
BVS141_25470	BVS141_25470	cysteine desulfurase	-1.4386	3.12E-02
BVS141_31660	BVS141_31660	aldo/keto reductase	-1.4812	2.67E-02

BVS141_29460	BVS141_29460	peptidase membrane zinc metallopeptidase	-1.4301	3.21E-02
BVS141_18580	BVS141_18580	CoA-binding protein	-1.4539	2.97E-02
yugS	BVS141_29450	Divalent ion export membrane protein	-1.3586	4.18E-02
nfrA	BVS141_36510	NfrA protein	-1.6178	1.59E-02

Table S3. List of Differentially Expressed Genes (DEGs) from up-regulated genes at P-value<0.05 and log_2FC>1

	Gene Name	Gene ID	Description	Log_2FC	P-value
Biosynthetic process	*carA*	BVS141_11560	carbamoyl phosphate synthase small subunit	2.0839	4.28E-03
	argJ	BVS141_11530	bifunctional ornithine acetyltransferase/N-acetylglutamate synthase protein	1.6808	1.90E-02
	argG	BVS141_27340	argininosuccinate synthase	1.8387	8.98E-03
	carB	BVS141_11570	carbamoyl phosphate synthase large subunit	2.0889	2.96E-03
	metE	BVS141_13690	5-methyl tetrahydropteroyl triglutamate/homocysteine S-methyltransferase	2.3601	7.68E-04
	metC	BVS141_12290	cystathionine beta-lyase	1.6171	2.36E-02
	BVS141_27330	BVS141_27330	argininosuccinate lyase	1.6870	1.45E-02
	argB	BVS141_11540	acetylglutamate kinase	2.9237	5.92E-04
	argF	BVS141_11580	ornithine carbamoyl transferase	2.3893	2.23E-03
Transport	*ydaO*	BVS141_04570	Uncharacterized amino acid permease YdaO	1.6158	1.61E-02
	pstS	BVS141_24130	Phosphate-binding protein pstS 1 PBP 1	2.0441	3.17E-03
	ycdI	BVS141_03090	zinc transport system ATP-binding protein	1.4361	4.89E-02
	opuAC	BVS141_03210	glycine betaine ABC transporter%2C glycine betaine-binding lipoprotein	1.4658	2.86E-02
	opuCC	BVS141_32170	osmoprotectant transport system substrate-binding protein	1.4698	2.83E-02
	BVS141_31880	BVS141_31880	cysteine ABC transporter permease	2.5313	1.55E-02
	opuBC	BVS141_32120	glycine betaine/carnitine/choline ABC transporter	3.4875	2.81E-03
	BVS141_32140	BVS141_32140	glycine/betaine ABC transporter ATP-binding protein	1.6496	3.84E-02
	BVS141_32160	BVS141_32160	amino acid ABC transporter permease	1.8235	7.49E-03
	opuAB	BVS141_03200	glycine betaine ABC transporter%2C permease OpuAB	1.3854	3.86E-02
	opuE	BVS141_07280	proline transporter	1.6078	1.70E-02
Signal peptide processing	*sipW*	BVS141_23790	SipW protein	5.8552	3.48E-02
Protein secretion, transformation and signal peptide processing	*tatA1*	BVS141_02910	Sec-independent protein translocase protein	3.8837	5.48E-03
	tatCD	BVS141_02920	component of the twin-arginine pre-protein translocation pathway	1.6399	2.31E-02
	comFB	BVS141_33810	ComF operon protein 2	1.4053	4.02E-02
	comC	BVS141_25980	membrane protease and transmethylase	1.8738	1.01E-02

	BVS141_11710	BVS141_11710	AppA protein	1.6381	1.75E-02
Metabolic process	*ydaE*	BVS141_04450	Probable D-lyxose ketol-isomerase	2.0822	2.55E-03
	yoaC	BVS141_19470	autoinducer 2 (AI-2) kinase	2.6509	1.03E-02
	glpQ	BVS141_02560	GlpQ protein	2.2992	1.35E-03
	ctpB	BVS141_33580	PDZ-containing carboxyl-terminal protease processing protease	1.8556	1.61E-02
	mtnB	BVS141_14050	methylthioribulose-1-phosphate dehydratase	1.3910	4.06E-02
	mtnX	BVS141_14040	2-hydroxy-3-keto-5-methylthiopentenyl-1-phosphate phosphatase	1.7386	1.37E-02
Gene regulation	*mutT1*	BVS141_04580	MutT/NUDIX family protein	1.6431	3.12E-02
	aag	BVS141_36970	3-methyladenine DNA glycosylase	5.8552	3.48E-02
	rpoZ	BVS141_16210	DNA-directed RNA polymerase subunit omega	1.5996	1.71E-02
	sigD	BVS141_17000	RNA polymerase sigma factor SigD	1.6362	1.54E-02
	rpsQ	BVS141_01590	30S ribosomal protein S17	1.3209	4.69E-02
	rpmC	BVS141_01580	50S ribosomal protein L29	1.3867	3.79E-02
	BVS141_00830	BVS141_00830	serine/threonine protein kinase%2C bacterial	1.9407	2.02E-02
Catabolic process	*ycgM*	BVS141_03520	proline dehydrogenase	1.7972	1.01E-02
	ysnA	BVS141_26350	nucleoside-triphosphatase	1.5555	2.58E-02
	BVS141_02980	BVS141_02980	lipase	5.4001	7.02E-06
	pel	BVS141_07880	pectate lyase	2.4215	2.05E-03
Protein folding and modification	*thrS*	BVS141_19330	Putative uncharacterized protein	2.0222	4.01E-02
	BVS141_24440	BVS141_24440	cytidine deaminase	1.3714	4.59E-02
Response to antibiotic	*BVS141_24970*	BVS141_24970	streptothricin acetyltransferase	2.8750	2.22E-02
Response to oxidative stress	*ohrB*	BVS141_13620	OhrB protein	2.1275	1.80E-03
Sporulation	*gerBC*	BVS141_34140	lipoprotein component of the germination receptor B	3.3481	2.80E-02
	gerBA	BVS141_34120	GerBA protein	4.1299	2.29E-03
	sspE	BVS141_08950	Small%2C acid-soluble spore protein gamma-type SASP	1.7507	2.75E-02
	ygxB	BVS141_09950	integral inner membrane protein	2.2324	8.73E-03
	spoIVFB	BVS141_25880	membrane metalloprotease	2.8678	9.11E-03

Flagellum organization	*fliR*	BVS141_16900	flagellar biosynthesis protein FliR	1.4933	2.61E-02
	fliQ	BVS141_16890	flagellar biosynthesis protein FliQ	2.1581	2.45E-03
	fliZ	BVS141_16870	Flagellar biosynthetic protein FliZ	1.5832	1.92E-02
	ylxF	BVS141_16780	FlaA locus 22.9 kDa protein ORF 6	1.8300	6.90E-03
	csrA	BVS141_33720	carbon storage regulator	1.9643	1.19E-02
	flhF	BVS141_16930	GTPase involved in the export of flagella	1.3249	4.83E-02
	BVS141_16940	BVS141_16940	flagellar biosynthesis protein	1.5471	2.18E-02
	fliJ	BVS141_16770	flagellar biosynthesis chaperone	1.7796	8.82E-03
	flgD	BVS141_16800	flagellar basal body rod modification protein	1.7442	9.92E-03
	fliY	BVS141_16850	flagellar motor switch protein	1.3955	3.65E-02
	fliM	BVS141_16840	flagellar motor switch protein FliM	1.4583	2.91E-02
Cell wall organization	*nagZ*	BVS141_02180	N-acetyl glucosaminidase lipoprotein	1.5299	4.62E-02
	tuaD	BVS141_33930	UDP-glucose 6-dehydrogenase TuaD	2.3163	1.07E-03
	BVS141_33910	BVS141_33910	Teichuronic acid biosynthesis protein TuaF	3.3560	2.70E-05
	tuaB	BVS141_33950	colanic acid exporter	2.1406	3.29E-03
	tuaC	BVS141_33940	glycosyltransferase	2.2063	2.48E-03
	tuaA	BVS141_33960	Sugar transferases involved in lipopolysaccharide synthesis TuaA	2.2633	7.78E-03
	tuaH	BVS141_33890	TuaH protein	2.3720	1.63E-03
	tuaE	BVS141_33920	Teichuronic acid biosynthesis protein TuaE	2.8191	1.26E-04
	tuaG	BVS141_33900	glycosyltransferase	3.0629	2.14E-04
	dltC	BVS141_36880	D-alanine-Poly (phosphoribitol) ligase subunit2	1.6159	1.76E-02
Chemotaxis	*cheA*	BVS141_16960	two-component sensor histidine kinase CheA	1.4084	3.48E-02
	cheY	BVS141_16860	two-component response regulator involved in modulation of flagellar	1.5154	2.40E-02
	cheB	BVS141_16950	chemotaxis-specific methylesterase	1.6434	1.58E-02
	cheW	BVS141_16970	chemotaxis protein CheW	1.5421	2.19E-02
Antibiotic biosynthetic process	*bacD*	BVS141_36120	BacD protein	1.7284	2.32E-02
	bacC	BVS141_36130	BacC protein	2.0233	8.55E-03

	srfAC	BVS141_03750	SrfAC protein	1.3461	4.53E-02
Peptidoglycan turnover	*yocH*	BVS141_19670	exported cell wall-binding protein	2.2453	1.02E-03
Proteolysis	*aprE*	BVS141_10820	serine alkaline protease	1.8421	7.53E-03
	BVS141_25290	BVS141_25290	Protease	1.5966	2.20E-02
Others	*BVS141_00320*	BVS141_00320	16S ribosomal RNA	1.3247	4.82E-02
	ydaP	BVS141_04590	pyruvate oxidase	1.3399	4.57E-02
	BVS141_09050	BVS141_09050	16S ribosomal RNA	1.3923	3.86E-02
	BVS141_06460	BVS141_06460	tRNA-Arg	1.3952	4.12E-02
	BVS141_38040	BVS141_38040	short chain dehydrogenase	1.4100	3.59E-02
	BVS141_35570	BVS141_35570	ABC transporter-like protein	1.4256	3.82E-02
	yfkH	BVS141_08340	tRNA-processing ribonuclease BN RNase BN	1.4305	4.49E-02
	BVS141_30320	BVS141_30320	conserved membrane protein of unknown function	1.4434	4.01E-02
	BVS141_30300	BVS141_30300	Uncharacterized membrane protein	1.4615	4.06E-02
	nhaX	BVS141_10220	stress response protein NhaX	1.4783	2.70E-02
	icaC	BVS141_08680	Biofilm PIA synthesis protein	1.4786	3.46E-02
	yxlA	BVS141_37120	Allantoin permease Allantoin transport protein	1.4832	3.74E-02
	ykrP	BVS141_13950	acyltransferases family protein	1.5212	4.93E-02
	yjlB	BVS141_12740	Putative uncharacterized protein with cupin domain	1.5331	2.51E-02
	BVS141_24930	BVS141_24930	transcriptional regulator	1.5417	2.22E-02
	BVS141_32520	BVS141_32520	Uncharacterized protein	1.5432	3.06E-02
	yqgC	BVS141_24170	integral inner membrane protein	1.5491	3.44E-02
	ywjC	BVS141_35560	Uncharacterized protein YwjC	1.5500	4.95E-02
	BVS141_30330	BVS141_30330	hypothetical protein	1.5518	2.20E-02
	BVS141_04680	BVS141_04680	YdaT protein	1.5674	2.31E-02
	BVS141_01140	BVS141_01140	16S ribosomal RNA	1.5676	1.96E-02
	yvaA	BVS141_31810	oxidoreductase%2C Gfo/Idh/MocA family	1.5739	2.50E-02
	BVS141_15020	BVS141_15020	chromosome partitioning protein	1.5745	2.53E-02
	ydjM	BVS141_06850	Putative uncharacterized protein YdjM/Rare lipoprotein A	1.5771	2.16E-02

BVS141_00210	BVS141_00210	tRNA-Ser	1.5957	2.56E-02
BVS141_02620	BVS141_02620	hypothetical protein	1.5997	2.67E-02
BVS141_12640	BVS141_12640	YjgB protein	1.6040	2.53E-02
BVS141_05160	BVS141_05160	tRNA-Leu	1.6091	3.38E-02
yycO	BVS141_38340	Uncharacterized protein YycO	1.6212	4.97E-02
yfkM	BVS141_08260	general stress protein 18	1.7247	1.06E-02
BVS141_05540	BVS141_05540	Mannose-6-phosphate isomerase	1.7349	3.95E-02
ybyB	BVS141_02540	Putative uncharacterized protein YbyB	1.7435	9.98E-03
yckC	BVS141_03650	integral inner membrane protein	1.7547	4.95E-02
yeeF	BVS141_07410	DNA binding protein	1.7718	1.38E-02
BVS141_10010	BVS141_10010	short chain dehydrogenase	1.7757	1.34E-02
ywdI	BVS141_36370	Uncharacterized protein YwdI	1.7831	2.98E-02
yqgZ	BVS141_23940	arsenate reductase	1.8008	7.78E-03
BVS141_19360	BVS141_19360	Uncharacterized protein	1.8146	1.28E-02
BVS141_10740	BVS141_10740	YhfH protein	1.8268	9.41E-03
yycD	BVS141_38650	Uncharacterized protein YycD	1.8360	4.91E-02
BVS141_06090	BVS141_06090	membrane protein MmpL3	1.8393	3.35E-02
rapI	BVS141_05390	response regulator aspartate phosphatase	1.8430	2.44E-02
ymzB	BVS141_17770	Uncharacterized protein YmzB	1.8537	6.39E-03
yocR	BVS141_19830	Sodium-dependent dopamine transporter DA transporter	1.8569	7.28E-03
BVS141_03380	BVS141_03380	ABC-type transport system permease component	1.8590	2.87E-02
yaaC	BVS141_00140	Putative uncharacterized protein YaaC	1.8776	1.70E-02
BVS141_04630	BVS141_04630	hypothetical protein	1.8947	5.78E-03
yocB	BVS141_19630	Putative Host attachment protein	1.9334	4.82E-03
norA	BVS141_38470	Quinolone resistance protein NorA	1.9506	9.25E-03
flbD	BVS141_16820	flagellar protein FlbD	1.9620	4.31E-03
yqeI	BVS141_24800	RNA-binding protein	1.9718	1.08E-02
BVS141_11830	BVS141_11830	conserved exported protein of unknown function	2.0588	2.82E-03

swrB	BVS141_17010	Swarming motility protein SwrB	2.0710	2.68E-03
dfnX	BVS141_22900	DfnX protein	2.0871	6.87E-03
yocK	BVS141_19700	General stress protein 16O GSP16O	2.1643	1.63E-03
ylyA	BVS141_15960	Putative DnaK suppressor related protein	2.2182	2.87E-02
ywsB	BVS141_34350	Cell wall-binding protein YwsB	2.3084	9.80E-04
BVS141_09970	BVS141_09970	alkaline phosphatase	2.3095	8.22E-04
sdaC	BVS141_05580	Threonine/serine transporter tdcCH(+)/threonine-serine symporter	2.3427	1.42E-02
BVS141_05140	BVS141_05140	tRNA-Gln	2.3427	2.88E-03
BVS141_20910	BVS141_20910	YpeQ protein	2.4527	5.20E-03
yyaD	BVS141_39170	integral membrane protein	2.4675	1.91E-02
yphE	BVS141_21800	Uncharacterized protein YphE	2.5801	2.32E-02
BVS141_04660	BVS141_04660	Uncharacterized protein	2.6083	3.53E-03
ywmE	BVS141_35080	Uncharacterized protein YwmE	2.7204	3.70E-04
gsiB	BVS141_04720	general stress protein GsiB	2.7960	6.60E-05
ydaD1	BVS141_04440	short chain dehydrogenase	2.8576	8.79E-05
BVS141_19770	BVS141_19770	hypothetical protein	2.9128	3.96E-03
ykzI	BVS141_15210	Uncharacterized protein YkzI	2.9704	2.88E-04
BVS141_36960	BVS141_36960	Uncharacterized protein	3.4723	1.98E-02
BVS141_36590	BVS141_36590	transglycosylase	3.5977	1.66E-06
phoD	BVS141_02900	alkaline phosphatase D	3.6753	1.08E-06
BVS141_20310	BVS141_20310	acetate CoA-transferase subunit A	3.7913	7.45E-03
BVS141_09620	BVS141_09620	HTH-type transcriptional repressor YtrA	3.8204	7.47E-04
csbD	BVS141_35030	CsbD protein	3.8407	4.66E-07
BVS141_05890	BVS141_05890	ribosomal RNA adenine dimethylase%2C phospholipid N-methyltransferase	3.8837	5.48E-03
yflT	BVS141_07870	General stress protein 17M GSP17M	3.9076	1.18E-07
ypzA	BVS141_20900	Uncharacterized protein YpzA	4.2034	1.74E-03
BVS141_09180	BVS141_09180	tRNA-Trp	4.3182	6.80E-05
ykvI	BVS141_14130	transporter	5.8552	3.48E-02

BVS141_15550	BVS141_15550	Uncharacterized protein	6.0447	2.19E-02
BVS141_27780	BVS141_27780	hypothetical protein	6.2122	1.41E-02
BVS141_28960	BVS141_28960	tRNA-Met	6.4982	6.15E-03
yrrI	BVS141_25360	permease	6.9414	1.41E-03

Table S4. List of expressed genes involved in secondary metabolites found in *Bacillus velezensis* S141 during *Cercospora canescens* inhibition at P-value<0.05 and $log_2FC>1$. The differentially expressed genes are indicate by * after gene name.

	gene name	Gene ID	Description	Log_2FC	P-value
**Antibiotic of polyketides**					
**Bacillaene**	*baes*	BVS141_17740	cytochrome P450 of bacillaene metabolism	1.1396	1.07E-01
	baeI	BVS141_17680	bacillaene biosynthesis enoyl-CoA hydratase	0.8379	2.55E-01
**Macrolactin**	*mlnA*	BVS141_15030	Malonyl CoA [acyl-carrier-protein] transacylase	-0.1768	8.60E-01
	mlnB	BVS141_15040	Macrolactin polyketide synthase MlnB	0.7988	2.38E-01
	mlnC	BVS141_15050	Macrolactin polyketide synthase MlnC	0.7963	2.58E-01
	mlnE	BVS141_15070	Macrolactin polyketide synthase MlnE	0.7362	2.75E-01
	mlnD	BVS141_15060	MlnD protein	0.8151	2.23E-01
	mlnF	BVS141_15080	MlnF protein	1.1101	1.32E-01
	mlnG	BVS141_15090	MlnG protein	0.6736	3.18E-01
	mlnH	BVS141_15100	MlnH protein	0.4976	4.97E-01
	mlnI	BVS141_15110	MlnI protein	0.3847	6.51E-01
**Difficidin**	*dnfB*	BVS141_22890	DfnB protein	0.9761	1.46E-01
	dfnC	BVS141_22880	DfnC protein	1.3513	5.85E-02
	dfnF	BVS141_22850	DfnF protein	0.6917	2.93E-01
	dnfG	BVS141_22840	DfnG protein	0.7436	2.58E-01
	dfnH	BVS141_22830	DfnH protein	0.6932	2.92E-01
	dfnI	BVS141_22820	DfnI protein	0.5622	3.92E-01
	dnfJ	BVS141_22810	DfnJ protein	0.7336	2.65E-01
	dfnK	BVS141_22800	Cytochrome P450 hydrlase	0.5264	4.30E-01
	dfnM	BVS141_22780	polyketide biosynthesis enoyl-CoA hydratase	0.1996	7.77E-01
	*dfnX**	BVS141_22900	DfnX protein	2.0871	6.87E-03
	dfnY	BVS141_22910	difficidin synthetase	0.7464	2.69E-01

**Non-ribosomal peptides**					
**Bacillomycin D**	*ycZE*	BVS141_03790	integral inner membrane protein regulating antibiotic production	-0.3196	6.68E-01
	bmyB	BVS141_18920	BmyB protein	1.2774	6.10E-02
	bmyD	BVS141_18950	BmyD protein	-0.9097	1.90E-01
	bmyC	BVS141_18910	BmyC protein	0.8296	2.19E-01
	bmyA	BVS141_18940	BmyA protein	0.8240	2.29E-01
**Bacilysin**	*bacA*	BVS141_36150	bacilysin biosynthesis protein%2C dehydratase	0.8705	3.26E-01
	bacB	BVS141_36140	Bacilysin biosynthesis protein BacB	0.6749	5.73E-01
	*bacC**	BVS141_36130	BacC protein	2.0233	8.55E-03
	*bacD**	BVS141_36120	BacD protein	1.7284	2.32E-02
	bacE	BVS141_36110	anticapsin/bacilysin excretion protein	0.3874	6.55E-01
	bacH	BVS141_36090	cyclohexenol-containin H4HPP in bacilysin synthesis	0.0655	9.22E-01
**Bacillibactin**	*ymfD*	BVS141_17360	bacillibactin exporter	0.4328	5.59E-01
	btr	BVS141_02160	transcriptional activator AraC/XylS of synthesis and uptake of the siderophore bacillibactin	0.0959	8.88E-01
**Surfactin**	*srfAC**	BVS141_03750	SrfAC protein	1.3461	4.53E-02
	srfAA	BVS141_03730	SrfAA protein	1.1047	9.76E-02
	srfAB	BVS141_03740	surfactin synthetase	1.0495	1.14E-01
	srfAD	BVS141_03760	SrfAD protein	0.9477	1.60E-01
